# Retraction: MicroRNA-410 Suppresses Migration and Invasion by Targeting MDM2 in Gastric Cancer

**DOI:** 10.1371/journal.pone.0269898

**Published:** 2022-06-08

**Authors:** The PLOS ONE Editors

Following the publication of this article [1], similarities were noted between this article and articles submitted by other research groups, including [2–10], of which one article [10] was previously retracted [11].

Similarities included the following figures, which appear to fully or partially overlap, despite being published in different articles and representing different conditions:

- scramble panel in Fig 2C of [1], and untreated and scramble panels in Fig 2D of [4].
- untreated panel in Fig 2D of [1], and control panel in Fig 4C of [2].
- inhibitor panel in Fig 2C of [1], and pCDNA-c-Myc + scramble panel in Fig 4C of [2].
- pCDNA-MDM2 + scramble panel in Fig 4C of [1], and untreat panel in Fig 3A of [3].
- pCDNA-MDM2 + miR-410 panel in Fig 4C of [1], and untreat and control panels in Fig 3B of [3].
- control and pCDNA + scramble panels in Fig 4C of [1], and mimic panel in Fig 3E of [10].

The corresponding author informed the journal that they obtained help to write and edit the manuscript. They also stated that informed consent was not obtained from all patients for the use of their tissue samples in this study. Due to this issue, the corresponding author requested that the article be retracted.

The concerns regarding similarities between the articles [1–10] remain unresolved and call into question the validity and provenance of the reported results, and the adherence of this article to the PLOS Authorship policy. Additionally, as the corresponding author stated that appropriate consent was not obtained from all patients, this article is not in compliance with the PLOS Human Subjects Research policy. In light of these issues, the *PLOS ONE* Editors retract this article [1].

HZ agreed with the retraction. All other authors either did not respond directly or could not be reached.
